# Report of an outbreak of toxicity from a novel drug of abuse in the UK: Eric-3

**DOI:** 10.1186/cc12205

**Published:** 2013-03-19

**Authors:** C Morden, S Haig, C Kelly

**Affiliations:** 1 Great Western Hospital, Swindon, UK

## Introduction

We present a case series of toxicity due to a novel substance in the UK: Eric-3. Novel drugs of abuse are becoming more common throughout the world, and they represent particular difficulties in their acute management. A recent report from the European Monitoring Centre for Drugs and Drug Addiction and Europol has reported 49 new psychoactive substances reported via its early warning system.

## Methods

This was a retrospective case-note review over a 6-month period. Patients were included if their presentation was due to recent ingestion of Eric-3. Physiological data, symptoms, outcome and destination of the patient from the emergency department were collected. Postmortem toxicological analysis was obtained for one of the two patients who died.

## Results

Forty-one attendances were identified from 18 patients. Two patients died and five were admitted to the ICU. Heart rate and temperature on arrival tended to be above normal (mean heart rate was 112 bpm, with an SD of 18; mean temperature was 37.45°C with an SD of 0.95). In total, 63.4% of attendances included agitation and 34.1% choreiform movements. α-Methyltryptamine and 3-/4-fluoroephedrine were found in the blood of one of the patients who died.

## Conclusion

In this outbreak in the UK, Eric-3 gave symptoms similar to other stimulants known as legal highs, including death. It may have been a novel substance, 3-/4-fluoroephedrine. This underlines the need for prospective data collection and early national and international information sharing.
